# An Estimate of Happiness among Students of Tehran University of Medical Sciences: A Means for Policy Making In Management of Health System

**Published:** 2011-11-01

**Authors:** F Farzianpour, M R Eshraghian, A H Emami, Sh Hosseini, S M Hosseini, D Farhud

**Affiliations:** 1 Department of Public Health, Tehran University of Medical Sciences, Tehran, Iran; 2 School of Medicine, Tehran University of Medical Sciences, Tehran, Iran; 3 Amir Kabir School of Electronic and Power, Tehran, Iran

**Keywords:** Happiness, University, Student, Medicine, Health, Iran

Dear Editor,

Happiness has several main components including the emotional component the presence of contentment and affection; the social component- the presence of good social relationships and the ability to receive support; and finally the cognitive component that causes the happy person to interpret information in a special way that leads to joy and optimism.[1][2][3][4][5]

Consequently, peoples’ assessment of themselves and their lives can include cognitive aspects- like judgments about life satisfaction- or emotional aspects like mood and affect.[5][6][7][8] Life satisfaction is defined as a level of personal awareness or more appropriately, a cognitive assessment of quality of life that can reflect a global assessment as well as one in such areas of life as family, self, etc.[6]

Several studies have pointed out the relationship between life satisfaction and some important factors like depression, hope and self-esteem. Additionally, life satisfaction is considered as a clear sign of successful adaptation with changes in life situations.[7] This is why adaptation in positive psychology has both positive and negative correlations- presence of life satisfaction and absence of mental illnesses.[6][7] In 2003, it was noted that a joyful environment is associated with higher life satisfaction and happiness. Environment in this sense can include country, city, neighborhood, household, and access to joyful tools (like music).[6]

The picture of mental and physical health is changing in Iran.[8] On a large scale, policy makers in health management pay considerably more attention to adolescents and youth and especially university students.[9] Developing an effective strategy for physical, mental, and social health of the youth constitutes one of the main policies in health management.[8][9]

The studied population was 224 students from Tehran University of Medical Sciences. We used two separate questionnaires in this study. The first was a researcher designed questionnaire that was tested in a pilot study of 15 students. This questionnaire has two parts. The first part contained personal and demographic data and satisfaction of students from the university. The second questionnaire was the Oxford Happiness Inventory (OHI). The first part contained 28 close ended or questions and for the second part, the international Argyle and Leu questionnaire was used that had 28 items and four choices.

There were 114 (50.9%) male students and 110 (49.1%) females. Students were chosen from seven faculties and 25 educational groups. 94.6% lived in urban areas, 59.4% were single, 33.9% were married, 49.1 percent lived with their families and owned a house (52.2%) and 33.9% of married students had one child. 27.7% had a sister and 27.2% had a brother. Employed students constituted 43.8%, of which 34.8% had governmental jobs. In the employed group, 1.4% were satisfied with their salary and 16.5% financially assisted their families. However, 13% received financial aid from their families. 83.5% of students were physically healthy, 96% were not addicted to any substance and 3.6% had a disability.

The answers to questions like “what is happiness?”(17.9% students answered, peace of mind), “what do you wish?” (19.2% students answered, easy life), “what problems do you have?”(29.9% students answered, financial), “Are you happy"? (45.1% students answered, yes), “Which picture describes you best?”(38.8% students answered the picture of a little happy one, No. 3; Table 1 and 2). Regarding students’ satisfaction for their university and educational group, 75.9% were satisfied with their choice of university.

**Table 1:** Frequency and percentage of happiness among students of Tehran University of Medical Sciences in 2010

**What is happiness?**	**No (%)**	**What do you wish?**	**No (%)**	**What problems do you have?**	**No (%)**	**Are you happy?**	**No (%)**
Peace of mind	40 (17.9)	Good job	3 (1.3)	Unemployment	34 (15.2)	Yes	101 (45.1)
Health	13 (5.8)	Adequate salary	4 (1.8)	Familial	13 (5.8)	No	18 (8.0)
Adequate salary	3 (1.3)	Easy life	43 (19.2)	Financial	67 (29.9)	Somehow	105 (46.9)
All of the above	167 (74.6)	All of the above	173 (77.2)	Physical	6 (2.7)	Total	224 (100.0)
Total	223 (99.6)	Total	223 (99.6)	Mental	16 (7.1)	-	-
Without answer	1 (4)	Without answer	1 (4)	Without answer	88 (39.3)	-	-
Total	224 (100.0)	Total	224 (100.0)	Total	224 (100.0)	-	-

**Table 2:** Frequency and percentage of following pictures describe feelings of students toward life in Tehran University of Medical Sciences in 2010.

**Completely happy**	**Happy **	**A little happy**	**Indifferent**	**A little sad**	**Sad**	**Completely sad **
**1**	**2**	**3**	**4**	**5**	**6**	**7**
No (%)	No (%)	No (%)	No (%)	No (%)	No (%)	No (%)
16 (7.1)	59 (26.3)	87 (38.8)	36 (16.1)	10 (4.5)	8 (3.6)	5 (2.2)

We used t-test and analysis of variance (ANOVA) to obtain a relation between happiness and other variables of the study. According to these tests, subjective happiness was not correlated with sex, field of education, type of residence, life expectation and physical health (p>0.05), but was correlated with university, marital status, living with parents, mental health, life problems, life satisfaction, face readings and career prospect (p<0.05).

The results of the studies showed that there was a significant correlation between happiness and life satisfaction and the economical, educational, cultural status of the family and also their attitude and believes towards life. Fairbank et al. reported that

happiness and life satisfaction came from social communication.[10] Galovski et al. showed that there was a correlation between happiness and the person's character.11 Gilman et al. believed that person's happiness was dependent on his adaptability to changes in his life.12 The research by Johnson et al. showed that having a satisfactory life, having no disease (being healthy) and an economically good life led to happiness.[13]

Since familial trauma affects the interactions between family members as evidenced by several studies- one can predict the possibility of divorce or severe conflicts in this group of parents. As parental conflicts can include different fields (educational, economical, and sexual…) it may not be valid to estimate their type and severity from children. Therefore, we consider only separation or living with parents in this study.[12][13][14] In 2006, Johnson and Krueger found a positive correlation between happiness and financial competence and income.[13]

Our study demonstrated no difference in happiness and life satisfaction between men and women. There were also no differences in the feeling of well-being between men and women across different educational groups. However, the difference between happiness across various faculties was significant. There was a significant difference between men and women in relation to happiness and socioeconomical, cultural, emotional, and mental conditions. Marital divorce and financial status were independent variables and happiness and life satisfaction were predictive variables; parental education was considered as the control variable. Furthermore, we failed to observe any difference among men and women in relation between happiness and needs, expectations and wishes. The mean±SD of happiness score in this study was 47.13±14.3 that is comparable to other studies in Iran. In comparison with studies performed after 1990, Argyle and Leu reported a 35.6 percent.[14] In studies done in England, USA, Canada and Australia, the scores varied between 38 and 42 %.[6]
